# 

**Published:** 1883-02

**Authors:** 


Official List of Changes of Stations and Duties of Medical Officers of the U. S. Marine-Hospital Service, October 1st. to December 31st. 1882.

Bailhache, P. H., Surgeon. Present detail continued until further orders, October 6, 1882. To proceed to Louisville, Ky., as inspector, October 13, 1882. Granted leave of sence for thirty days, November 10, 1882.

Vansant, John, Surgeon. Granted leave of absence for twenty days, November 18, 1882.

Hutton, W. H. H., Surgeon. To proceed to Louisville, Ky., and assume charge of the Service, October 7 and 14, 1882.

Miller, T. W., Surgeon. To continue at present station until further orders, October 6, 1882.

Wyman, Walter. To inspect keepers and crews of the Life Saving Service, October 5, 1882.

Long, W. H., Surgeon. To proceed to Detroit, Mich., and assume charge of the Service, October 7 and 14, 1882.

Murray, R. D., Surgeon. Having returned from service in the yellow-fever epidemic in Texas, to report in person to the Surgeon General, M. H. S., December 4, 1882. Granted leave of absence until February 28, 1883, December 19, 1882.

Fessenden, C. S. D., Surgeon. To proceed to St. Louis, Mo., and assume charge of the Service, October 7, 1882.

Purviance, George, Surgeon. To inspect keepers and crews of the Life Saving Service, October 21, 1882.

Sawtelle, H. W., Surgeon. To proceed to New York, N. Y., and assume charge of the Service, October 7, 1882.

AUSTIN, H. W., Surgeon. To inspect keepers and crews of the Life Saving Service, October 5, 1882.

FISHER, J. C., Passed Assistant Surgeon. Present detail continued until further orders, October 6, 1882. To proceed to Alexandria, Va., as inspector, October 21, 1882.

HEATH, W. H., Passed Assistant Surgeon. Granted leave of absence for fourteen days, December 28, 1882.

PORTER, F. D., Passed Assistant Surgeon. To inspect keepers and crews of the Life Saving Service, October 5, 1882. To proceed to Evansville, Ind., for temporary duty, November 21, 1882. To proceed to Charleston, S. C., and assume charge of the Service, December 21, 1882.

O'CONNOR, F. J., Assistant Surgeon. To proceed to Norfolk, Va., for temporary duty, October 14, 1882. To rejoin his station (Detroit), November 4, 1882.

WHEELER, W. A., Assistant Surgeon. Relieved of duty at Charleston, S. C., and placed on waiting orders, December 22, 1882.

ARMSTRONG, S. T., Assistant Surgeon. To examine keepers and crews of the Life Saving Service, October 5, 1882.

BENNETT, P. H., Assistant Surgeon. To examine keepers and crews of the Life Saving Service, October 5, 1882.

AMES, R. P. M., Assistant Surgeon. Granted leave of absence for twenty-one days, November 23, 1882.

DEVAN, S. C., Assistant Surgeon. To examine keepers and crews of the Life Saving Service, October 13, 1882. To inspect unserviceable property at the San Francisco Marine Hospital, October 20, 1882.

KALLOCH, P. C., Assistant Surgeon. To inspect keepers and crews of the Life Saving Service, October 5, 1882.

CORRECTION.—Owing to an unfortunate error in the January number, the following selected articles appeared without proper credit to our exchanges in which they originally appeared :

"Locomotor Ataxia, Case," by A. M. Carpenter, from the *Indiana Med. Reporter.*

CLINICS.

MONDAY.

Eye and Ear Infirmary—1.15 to 2.15 p. m., Otological, by Dr. Schaefer; 2.15 to 3.30 p. m., Ophthalmological, Dr. Holmes.

Mercy Hospital—2 p. m., Medical, Profs. Hollister and Quine.

Rush Medical College—3 p. m., Dermatological and Venereal, by Prof. Hyde.

Woman's Medical College—2 p. m., Dermatological and Venereal, by Prof. Maynard.

TUESDAY.

Cook Co. Hospital—2 to 4 p. m., Medical and Surgical Clinics.

Mercy Hospital—2 p. m., Surgical Clinic, by Prof. Andrews.

Woman's Medical College—10 a. m., Prof. Ingals.

WEDNESDAY.

Chicago Medical College—2 p. m., Eye and Ear, by Prof. Jones.

Rush Medical College—2 p. m., Medical by Prof. Bridge; 3 p. m., Ophthalmological and Otological, by Prof. Holmes: 3 to 4 p. m., Diseases of the Chest, by Prof. Ross.

Woman's Medical College—2 p. m., Eye and Ear, by Dr. W. T. Montgomery; 3 p. m., Diseases of Children, by Prof. Chas. W. Earle.

Eye and Ear Infirmary—2.30 p. m.. Dr. E. J. Gardiner.

THURSDAY.

Chicago Medical College—2 p. m., Gynæcological, Prof. Dudley.

Rush Medical College—2 p. m., Diseases of Children, by Prof. Knox; 3 p. m., Diseases of the Nervous System, by Prof. Lyman.

Eye and Ear Infirmary—1.15 to 2.25 p. m., Otological. by Dr. Schaefer; 2.15 to 3.30, p. m., Ophthalmological, Dr. Holmes.

Woman's Medical College—3 p. m., Surgical, by Prof. Owens.

College of Physicians and Surgeons—2 p. m., Medical, by Prof. S. A. McWilliams; 3 p. m., Surgical, by Prof. R. L. Rea.

FRIDAY.

Cook County Hospital—2 to 4 p. m., Medical and Surgical Clinics.

Mercy Hospital—2 p. m., Medical, by Prof. Davis.

SATURDAY.

Rush Medical College—2 p. m., Surgical, by Prof. Gunn.

Mercy Hospital—2 p. m., Surgical Clinic, by Prof. Andrews.

Chicago Medical College—3 p. m., Neurological, Prof. Jewell.

Woman's Medical College—2 p. m., Gynæcological, by Prof. T. D. Fitch.

College of Physicians and Surgeons—2 p. m., Diseases of the Chest, by Prof. S. A. McWilliams; 3 p. m., Gynæcological, by Prof. A. Reeves Jackson.

Daily Clinics, from 2 to 4 p. m., at the Central Free Dispensary, at the South Side Dispensary and at the West Side Dispensary.

